# Action potential initiation in a two-compartment model of pyramidal neuron mediated by dendritic Ca^2+^ spike

**DOI:** 10.1038/srep45684

**Published:** 2017-04-03

**Authors:** Guosheng Yi, Jiang Wang, Xile Wei, Bin Deng

**Affiliations:** 1 School of Electrical and Information Engineering, Tianjin University, Tianjin 300072, China

## Abstract

Dendritic Ca^2+^ spike endows cortical pyramidal cell with powerful ability of synaptic integration, which is critical for neuronal computation. Here we propose a two-compartment conductance-based model to investigate how the Ca^2+^ activity of apical dendrite participates in the action potential (AP) initiation to affect the firing properties of pyramidal neurons. We have shown that the apical input with sufficient intensity triggers a dendritic Ca^2+^ spike, which significantly boosts dendritic inputs as it propagates to soma. Such event instantaneously shifts the limit cycle attractor of the neuron and results in a burst of APs, which makes its firing rate reach a plateau steady-state level. Delivering current to two chambers simultaneously increases the level of neuronal excitability and decreases the threshold of input-output relation. Here the back-propagating APs facilitate the initiation of dendritic Ca^2+^ spike and evoke BAC firing. These findings indicate that the proposed model is capable of reproducing *in vitro* experimental observations. By determining spike initiating dynamics, we have provided a fundamental link between dendritic Ca^2+^ spike and output APs, which could contribute to mechanically interpreting how dendritic Ca^2+^ activity participates in the simple computations of pyramidal neuron.

Pyramidal neurons are common cell types found in the cerebral cortex and hippocampus of mammalian brain^1^,^2^,^3^. Their structures are characterized by a pyramidal shaped soma and extended apical and basal dendritic trees. This kind of nerve cells have powerful capability of processing information, which could effectively and precisely transform incoming signals into specific patterns of action potential (AP) output. During this procedure, their dendrites play a particularly vital role, since they are the predominant receiving sites for synaptic signals^1^,^4^,^5^,^6^,^7^,^8^. The vast branches of dendritic tree endow a pyramidal cell with distinctive morphological feature, which disperse the primary input locations. It is known that APs usually occur in the initial segment of the axon. Due to such spatial arrangement, the apical dendrites have to deliver input signals to the site of AP initiation. Their function is not solely to receive information from connected input cells and transmit it to the axon. Each dendritic branch is also a basic signalling unit for integrating synaptic inputs^4^,^6^,^7^,^8^,^9^,^10^,^11^, which determines how the receiving signals propagate to the axon. Such nonlinear integration operated by dendrites has a profound influence on neuronal and cortical computation^1^,^2^,^4^,^5^,^6^,^7^,^8^,^9^,^10^.

The dendrites of pyramidal cells rely on their intrinsic nonlinearities, including voltage-gated channels and complex morphology, to integrate synaptic signals^4^,^5^,^6^,^7^,^8^,^9^,^10^,^11^. The active ionic channels in their apical dendrites are particularly important in synaptic integration. A common channel is the voltage-dependent Ca^2+^ current that flows into the cell^1^,^4^,^5^,^6^,^7^,^12^,^13^,^14^. The activation of its conductance could cause a threshold-dependent, all-or-none regenerative response in dendrites, which is often referred to as dendritic Ca^2+^ spike^4^,^7^,^11^,^14^,^15^,^16^,^17^. The existence of active Ca^2+^ channel in apical dendrites make pyramidal neurons operate in either global or two-stage integration mode^18^,^19^. For simple global integration mode, input signals directly contribute to AP output by triggering excitatory postsynaptic potentials (EPSPs) that spread to the AP initiation zone. In latter integration mode, the synaptic input directly activates the Ca^2+^ channel in dendrites and triggers dendritic spikes^7^,^14^,^15^,^16^,^17^,^20^, which propagates forward to the axon where the global integration occurs^18^,^19^. Such integration lies at the heart of neural computation, which is tightly related to coincidence detection^1^,^16^,^21^,^22^, orientation tuning^22^, binding of synaptic signals from brain areas^23^, and enhancing stimulus selectivity^24^. Understanding how it participates in AP output is therefore fundamental to understanding how relevant circuits function in cortical computation of mammalian brain.

Earlier studies have extensively explored the dendritic Ca^2+^ activities and their effects on neuronal firing behaviors with *in vitro* approaches. It is found that the synaptic inputs at different sites of dendrite^16^,^17^,^25^, the back-propagating APs^17^,^26^,^27^, and the local NMDA spikes^7^,^28^,^29^ are all important determinants for activating Ca^2+^ conductance and triggering dendritic Ca^2+^ spike. This regenerative event at apical dendrites can boost distal synaptic inputs and enhance synaptic efficacy, which is hypothesized as the main biological mechanism for propagating synaptic inputs at the distal tuft to the soma of layer 5 pyramidal neurons^15^,^16^,^17^,^28^. It is usually characterized by a steep change followed by a plateau in the subthreshold input-output transformations conferred by dendrites. Further, the additional inward current associated with Ca^2+^ spike provides a strong local depolarization in dendritic membrane, which can enhance the somatic/axonal AP outputs. In particular, it can significantly increase the gain of input-output relation of pyramidal neuron, which triggers a burst of APs in the soma/axon and switches the firing mode of the cell to bursting^23^,^26^,^30^,^31^,^32^. However, it is still not well understood how dendritic Ca^2+^ spike participates in AP initiation to influence the somatic/axonal output.

In addition to above *in vitro* observations, there are also modeling studies that focus on the dendritic Ca^2+^ activity^21^,^33^,^34^,^35^,^36^,^37^. Most of them use biophysically realistic neurons that are modeled in NEURON or GENESIS to understand the mechanism underlying the generation and propagation of dendritic Ca^2+^ spike. Such complex multi-compartment models are not sufficiently simple to allow one to uncover the dynamical or biophysical basis for AP initiation related to dendritic Ca^2+^ activity. There are also studies that use two compartments to model pyramidal neurons with dendritic Ca^2+^ channel. Such simple point-neuron models have been adopted to study their firing patterns^38^,^39^,^40^, spike-timing predictions^41^, spike timing-dependent plasticity^42^, and spike-frequency adaptation^43^. However, it has attracted little attention about the somatic/axonal AP initiation associated with dendritic Ca^2+^ spike. Further, Larkum *et al*.^26^ have used a two-compartment integrate-and-fire (IF) model to reproduce the gain modulation of pyramidal cell induced by top-down dendritic Ca^2+^ spike. However, in IF model an AP is explicitly generated when its membrane voltage reaches a predefined threshold^44^. That is, the IF model is unable to reproduce how inward and outward ionic currents interact at the subthreshold potentials to initiate AP. Therefore, it is still largely unknown that how dendritic Ca^2+^ spike affects the AP initiation of individual pyramidal cells.

Here we develop a five-dimensional (5D) two-compartment model (as shown in Fig. 1a) by introducing Ca^2+^ current into the passive dendrite of a reduced Pinsky-Rinzel (PR) model^45^,^46^. Contrary to IF model, the generation of APs in our model implicitly results from the dynamical interactions of inward Na^+^ and outward K^+^ currents at the subthreshold voltages^45^,^46^,^47^. With this model, we have systematically investigated the firing behaviors of the pyramidal neuron with passive and active dendrite to the input current injected at different sites of the neuron. The dynamical basis for relevant AP initiation is determined with phase plane and bifurcation analysis. Our simulations indicate that the proposed model here is able to reproduce a variety of *in vitro* experimental observations of pyramidal neurons, which is also amenable to both dynamical analysis and efficient simulation.

## Results

### Somatic input is unable to trigger dendritic Ca^2+^ spike in the absence of dendritic input

**Figure 1:**
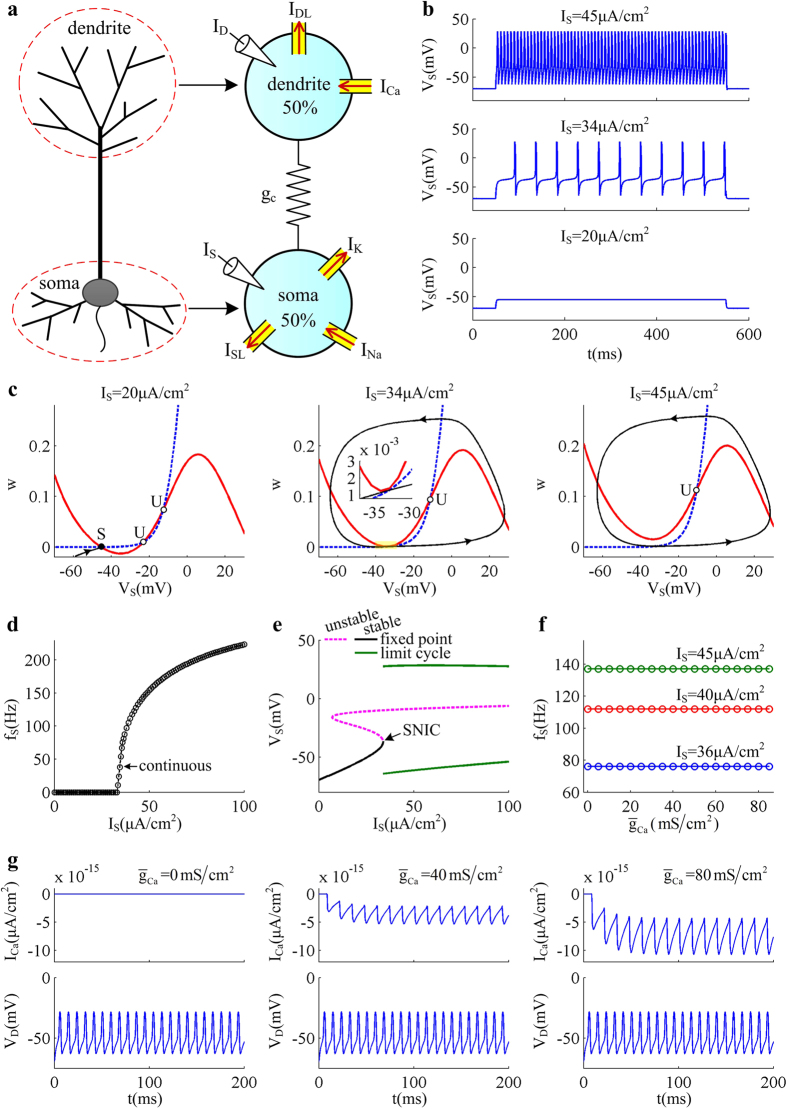
Input-output relation and relevant spike initiating dynamics evoked by somatic input. (**a**) 5D two-compartment model of the pyramidal neuron. One chamber represents the basal zone around soma, and the other one is apical dendrite. Two chambers are connected by an internal conductance g_c_. The stimulus current can be independently injected into soma and dendrite. Red arrows in the right panel indicate the direction of relevant current flow. (**b**) Sample responses triggered by three values of *I*_S_, which is indicated on the top of each panel. (**c**) Phase portraits in 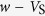 plane to subthreshold and suprathreshold *I*_S_. Blue dotted line represents *w*-nullcline and red solid line is *V*_S_-nullcline. They represent the states where corresponding variable neither increases nor decreases. Black solid line is a sample *V*_S_ trajectory, where arrows indicate the direction of its motion. ‘S’ indicates the intersection between two nullclines is stable and ‘U’ is unstable. **(d)** Relation between average firing rate *f*_S_ and somatic input *I*_S_, i.e., 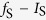 curve. **(e)** Bifurcation diagram of the 5D model, here the bifurcation parameter is *I*_S_. Vertical axis gives the somatic voltage *V*_S_ at the fixed point or at the max/min of limit cycle as input *I*_S_ is increased. (**f)** Relation between average firing rate *f*_S_ and Ca^2+^ conductance 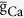 (i.e., 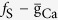 curve) with three values of input *I*_S_. **(g)** Time courses of dendritic Ca^2+^ current *I*_Ca_ and dendritic voltage *V*_D_ in the cases of 
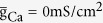
, 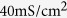, and 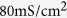. Corresponding somatic injection is 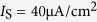. For **(b**–**g)**, the dendritic input is 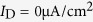.

We first investigate the spiking properties of the 5D model neuron to somatic input *I*_S_. The current *I*_D_ injected at dendritic chamber is absent, i.e., 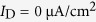. Figure 1b–e give the input-output relation and corresponding dynamical basis of AP initiation in the case of 
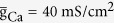
. We find that the neuron is unable to generate APs when 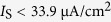 (Fig. 1b and d). At these small values of *I*_S_, the neuron exist in quiescent state and its somatic membrane potential *V*_S_ is eventually stabilized at a subthreshold voltage. With 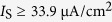, the neuron generates repetitive APs. In this case, the average firing rate *f*_S_ increases with input *I*_S_ from 0 Hz. The relation between *f*_S_ and *I*_S_ (i.e., *f*_S_ − *I*_S_ curve) is continuous (Fig. 1d).

Figure 1c illustrates the dynamical basis of AP initiation associated with the observed behaviors shown in Fig. 1b and d. With 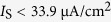, the *V*_S_- and *w*-nullclines intersect at three points in 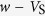 phase plane (left panel, Fig. 1c). Since the leftmost intersection is a stable node, all of *V*_S_ trajectories converge to this equilibrium and the neuron does not fire APs. Increasing *I*_S_ shifts *V*_S_-nullcline upwards, while does not alter the position of *w*-nullcline. In this case, the distance between stable node and unstable saddle decreases. With 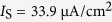, these two equilibriums coalesce and annihilate each other. At the same time, a stable limit cycle is generated in 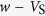 phase plane (Fig. 1c). Since the stable node corresponding to resting state no longer exists, *V*_S_ trajectory jumps to the limit cycle attractor and the neuron starts to fire tonic spikes. The transition from resting to repetitive spiking occurs through a saddle-node on invariant circle (SNIC) bifurcation of equilibrium (Fig. 1e), which corresponds to the continuous 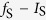 curve.

In the absence of *I*_D_, the firing rate *f*_S_ of the model evoked by *I*_S_ does not change with the maximum conductance 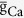 of dendritic Ca^2+^ current (Fig. 1f). With 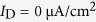, there is no external driver activating Ca^2+^ conductance and thus dendritic Ca^2+^ spike is missing. Here *I*_S_ only activates the Na^+^ channel in soma. Although the Na^+^-APs can be back-propagated to dendrite, such bottom-up input is unable to drive *V*_D_ to reach the threshold voltage for activating Ca^2+^ current (Fig. 1g). Under these conditions, increasing 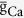 produces no effects on the spiking behaviors of the neuron to somatic input.

### Apical input activates dendritic Ca^2+^ current and results in burst of somatic APs

**Figure 2:**
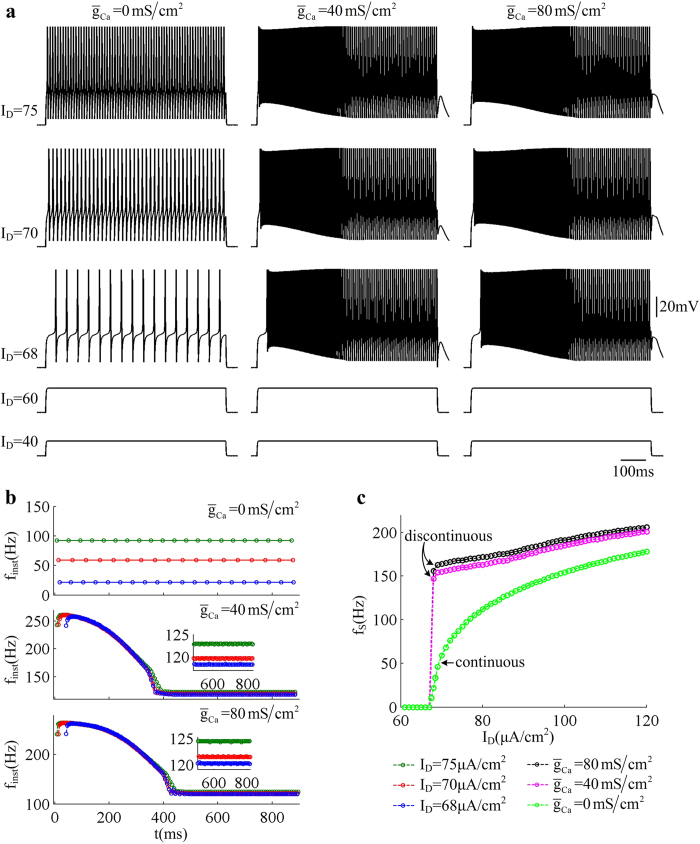
Firing properties evoked by dendritic input. (**a**) Sample responses of the 5D model triggered by apical input *I*_D_ in the cases of 
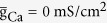
, 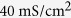, and 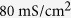. *I*_D_ is in 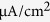, which is indicated on the left. **(b)** Instantaneous firing rate *f*_inst_ with three values of 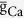. Corresponding dendritic inputs are 
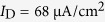
, 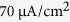, and 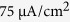. (**c**) Relation between average firing rate *f*_S_ and input *I*_D_ (i.e., 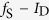 curve) with three values of 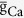. Somatic injection is 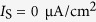.

In this section, we investigate how 5D model neuron responds to apical input injected at dendrite. Here somatic input *I*_S_ is absent, i.e., 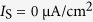. The firing behaviors of the model is determined with different values of 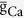, which are summarized in Fig. 2.

With 
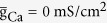
, the dendritic Ca^2+^ current is blocked and the dendrite becomes passive. In this case, the neuron exists in quiescent state with 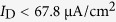 and there are still no APs generated. Once *I*_D_ exceeds 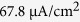, repetitive APs are initiated in somatic chamber (left panel, Fig. 2a). The threshold of *I*_D_ here is much larger than that of *I*_S_ directly injected to soma. This is because the dendritic compartment in the model serves as a current sink allowing only part of the input current to invade the soma. From Fig. 2c, one can find that the *f*_S_ − *I*_D_ curve is continuous when blocking dendritic Ca^2+^ current, which indicates that the neuron is able to fire low-frequency APs to injected current *I*_D_. As *I*_D_ is increased, the slope of *f*_S_ − *I*_D_ curve (i.e., input-output gain) is obviously reduced. Further, the evoked spike trains are always repetitive and the instantaneous firing rate *f*_inst_ remains constant with time (top panel, Fig. 2b). The *f*_inst_ in our study is calculated based on the reciprocal of relevant ISI in each spike train.

With 
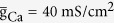
 or 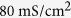, there is active Ca^2+^ current in dendritic chamber. We find that the threshold value of *I*_D_ for triggering somatic APs does not change with 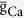, which is still 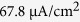. However, the time course of the spike train is no longer repetitive (center and right panels, Fig. 2a). At the onset of *I*_D_, the model generates a burst of high-frequency spikes, which then slowly transits to repetitive spiking with low frequency. From Fig. 2b, one can observe that the instantaneous firing rate *f*_inst_ first quickly increases to a peak value and then slowly decays to a lower plateau level. Under these conditions, the *f*_S_ − *I*_D_ curve with 
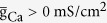
 is no longer continuous, and the neuron becomes unable to maintain low-frequency spiking. As shown in Fig. 2c, the average firing rate *f*_S_ immediately jumps to a high value once *I*_D_ reaches the threshold for triggering APs. After that, the slope of 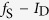 curve (i.e., input-output gain) with 
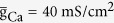
 or 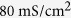 both changes little as *I*_D_ is increased. These simulations indicate that the activation of dendritic Ca^2+^ current boosts excitatory input *I*_D_ and facilitates the generation of somatic APs, i.e., an active dendritic integration occurs.

### Dynamical basis for the burst of APs associated with dendritic Ca^2+^ spike

In previous section, we have determined the spiking properties of 5D model neuron stimulated by apical input *I*_D_ alone. Our next step is to uncover the AP initiating dynamics associated with these behaviors.

**Figure 3:**
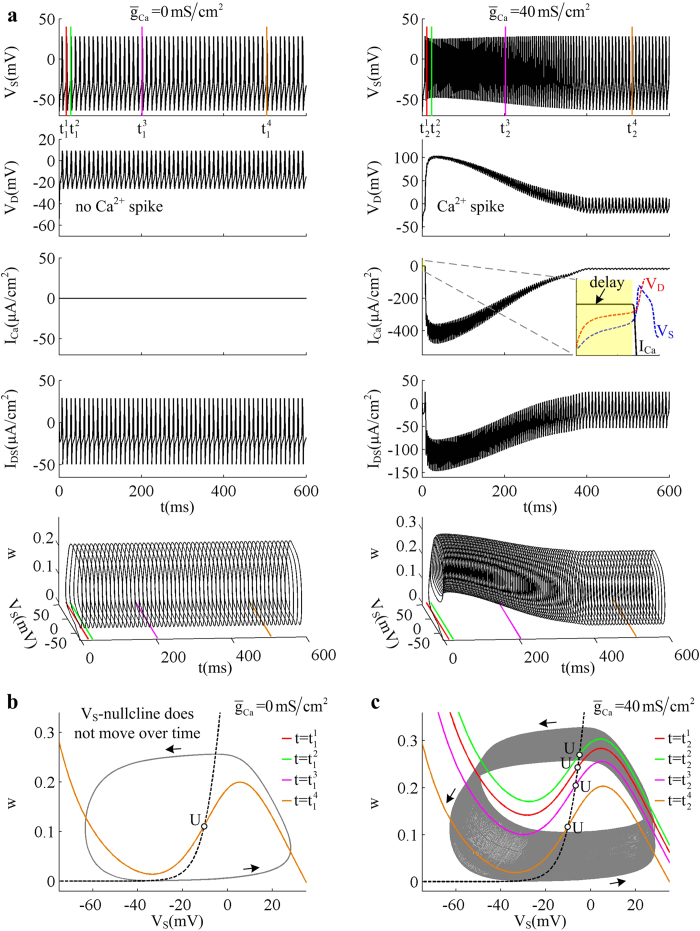
Spike initiating dynamics evoked by dendritic input. (**a**) Sample responses of the 5D model with 
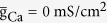
 and 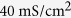. Somatic voltage *V*_S_, dendritic voltage *V*_D_, dendritic Ca^2+^ current *I*_Ca_, and internal current *I*_DS_ are plotted against time. The close-up shows that the Ca^2+^ conductance is activated when dendritic voltage *V*_D_ exceeds a threshold value. The sample response is also plotted in 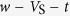 space (bottom panel). Color lines indicate the times at which the nullclines in **(b)** and **(c)** are calculated. Here dendritic input is 
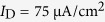
. **(b)** Two-dimensional phase portraits in 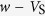 plane for 
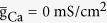
. **(c)** Phase portraits in 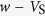 plane for 
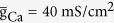
. For **(b)** and **(c)**, the *V*_S_- and *w*-nullclines are respectively calculated at four time points indicated by colored lines in (**a**). Black dotted lines represent *w*-nullclines, which are the same at different time points. The inverted N-shape lines with other colors are the *V*_S_-nullcline at the corresponding time point indicated in **(a)**. With 
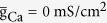
, the *V*_S_-nullcline does not move over time. Gray solid line is the sample *V*_S_ trajectory of the recorded spike trains, and black arrows indicate the direction of its motion. ‘U’ indicates unstable fixed point. Somatic injection is 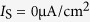.

When blocking dendritic Ca^2+^ current (i.e., 
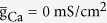
), the dendrite becomes passive. Its membrane voltage *V*_D_ only oscillates repetitively along with *V*_S_ and there are no dendritic spikes evoked (left panels, Fig. 3a). Here the internal current *I*_DS_ between two chambers is also repetitive, which transmits depolarizing input *I*_D_ from dendrite to soma. Under these conditions, the intersection between *V*_S_- and *w*-nullclines and corresponding limit cycle attractor both remain unchanged from one AP to the next (Fig. 3b). As a result, the neuron generates repetitive spike trains.

With 
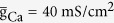
, the onset of apical input *I*_D_ depolarizes membrane voltage *V*_D_ and *V*_S_. When *V*_D_ exceeds a threshold value, the Ca^2+^ conductance is activated and then a broader Ca^2+^ spike is initiated in the dendrite (right panels, Fig. 3a). When this event occurs, *I*_Ca_ first rapidly falls to a minimum value and then slowly rises to a steady-state plateau level. Note that the negative sign of *I*_Ca_ means this current is inward. Since Ca^2+^ flows into the dendritic cell, such Ca^2+^ spike evokes a prolonged obvious depolarization of dendritic membrane voltage. Under these conditions, there is an obviously depolarizing sink in internal current *I*_DS_, which coincides with dendritic Ca^2+^ spike. That is, the presence of dendritic Ca^2+^ spike boosts apical input as it spreads to soma. Due to such active integration, a constant input of 
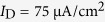
 is amplified to a non-periodic current *I*_DS_ with a maximum intensity around 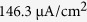 to invade the soma. Such depolarizing internal current shifts *V*_S_-nullcline upwards instantaneously in the first rapid phase of Ca^2+^ spike, and forces two nullclines to interact at an unstable fixed point (Fig. 3c). Then all of the *V*_S_ trajectories converge to limit cycle attractor, and the neuron generates initial APs. Before *I*_Ca_ reaches peak value, the amplitude of depolarizing *I*_DS_ continues to increase. It drives unstable fixed point upwards and the limit cycle attractor moves upwards accordingly, which makes firing rate *f*_inst_ further increase. In the second phase of dendritic Ca^2+^ spike, *I*_Ca_ slowly gets weak and the amplitude of depolarizing *I*_DS_ becomes to decrease. In this case, the unstable fixed point and relevant limit cycle attractor both moves downwards (Fig. 3c), which leads to a decay of firing rate *f*_inst_ to its steady-state plateau level. Therefore, the model generates a burst of somatic APs when dendritic Ca^2+^ spike is initiated. Once such local spike is evoked, the amplitude of dendritic voltage *V*_D_ and internal current *I*_DS_ with a specific value of 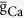 both varies little with apical input *I*_D_. Then, the input-output gain with 
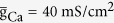
 or 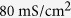 changes little in the observed range of *I*_D_.

**Figure 4:**
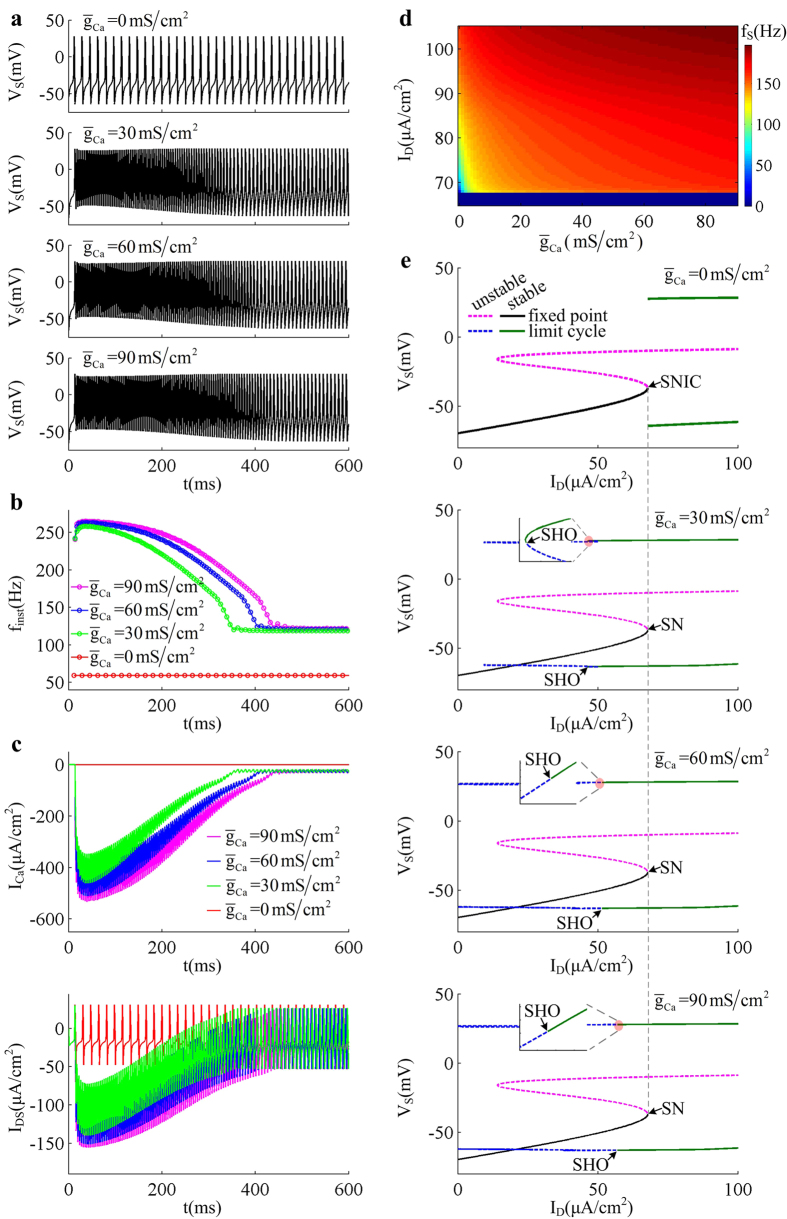
Effects of varying Ca^2+^ conductance on spike initiating dynamics evoked by dendritic input. (**a**) Spike train recorded in the soma of 5D model in the cases of 
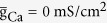
, 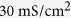, 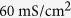, and 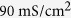. (**b**) The instantaneous firing rate *f*_inst_ for each spike train. (**c**) Time courses of dendritic Ca^2+^ current *I*_Ca_ and corresponding internal current *I*_DS_ with each value of 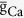. For **(a**–**c**), the corresponding dendritic input is 
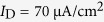
. (**d**) Average firing rate *f*_S_ is plotted against *I*_D_ and 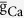. (**e**) Bifurcation diagram with different values of 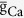. Here the bifurcation parameter is apical input *I*_D_. Type of the bifurcation for equilibrium or limit cycle is indicated on each plot. Somatic input is 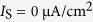.

Figure 4 shows the AP initiating dynamics associated with the firing behaviors of the neuron as Ca^2+^ conductance 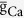 is increased from 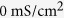 to 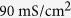. Increasing 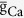 results in more somatic APs during the course of dendritic Ca^2+^ spike (Fig. 4a and b). It is known that the intensity of *I*_Ca_ is proportional to 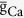, and increasing its conductance extends the duration of dendritic Ca^2+^ spike (Fig. 4c). Then, the depolarizing current *I*_DS_ induced by dendritic spike becomes progressively more prominent with 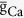. Such stronger depolarizing current accelerates the spike initiation in somatic chamber, and drives neuron to fire more APs at a given value of *I*_D_. Then, the average firing rate *f*_S_ increases as Ca^2+^ conductance is increased (Fig. 4d). However, varying 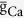 does not alter the kinetics or voltage-dependency of Ca^2+^ current. Then, the threshold of apical input *I*_D_ for activating dendritic Ca^2+^ channel or evoking somatic APs remains unchanged as 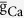 is increased (Fig. 4d). From Fig. 4e, one can find that blocking dendritic Ca^2+^ current results in a SNIC bifurcation of the equilibrium, which endows the neuron with a continuous 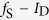 curve (Fig. 2c). Introducing dendritic Ca^2+^ channel extends the stable limit cycle to the value of *I*_D_ below the bifurcation point of equilibrium, followed by unstable limit cycle. In this case, the equilibrium loses its stability via a saddle-node (SN) bifurcation, which corresponds to the transitions of resting to tonic spiking. When such kind of bifurcation occurs, the neuron fails to fire low-frequency APs^48^, and its 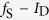 curve becomes discontinuous (Fig. 2c). Unlike equilibrium, the limit cycle transits from unstable to stable via saddle homoclinic orbit (SHO) bifurcation (Fig. 4e). As *I*_D_ is increased, the unstable limit cycle disappears via a subcritical SHO bifurcation, and the stable limit cycle appears via a supercritical SHO bifurcation. The presence of Ca^2+^ current in dendrite increases the dimension of the system, and makes the SHO bifurcations of limit cycle attractor occur at the value of *I*_D_ below SN bifurcation. What’s more, the SHO bifurcation also occurs at different values of apical input as 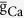 is increased (Fig. 4e). All these modulations of firing behavior and corresponding spike initiating dynamics with 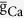 are due to dendritic Ca^2+^ activity, which endows dendrites with the powerful ability to actively integrate excitatory inputs.

### Coincident somatic and dendritic inputs facilitate dendritic Ca^2+^ spike

**Figure 5:**
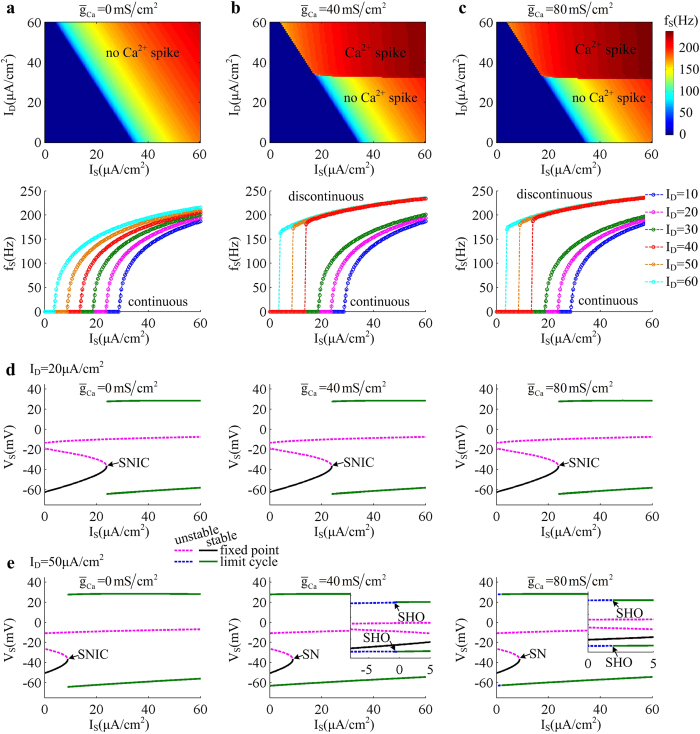
Firing properties evoked by coincident somatic and dendritic inputs. The firing rate *f*_S_ of the 5D model is calculated for three values of 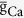, which are **(a**) 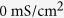. **(b**) 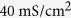 and **(c**) 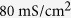, respectively. For **(a–c)**, top panels give the average firing rate *f*_S_ plotted against input *I*_S_ and *I*_D_. Bottom panels show the 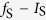 curves with different values of *I*_D_. The bifurcation diagrams related to coincident inputs at soma and dendrite with each value of 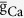 are given in **(d)** and **(e)**. Here the bifurcation parameter is somatic input *I*_S_. We compute one-parameter bifurcation with two values of dendritic input, which are **(d)** 
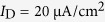
 and **(e**) 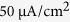. The lower value of *I*_D_ is unable to activate dendritic Ca^2+^ conductance, but the latter could trigger dendritic Ca^2+^ spike. Type of the bifurcation for equilibrium or limit cycle is indicated on each plot.

Here we determine the firing properties of the 5D model neuron evoked by the stimulation of coincident *I*_S_ and *I*_D_, which are summarized in (*I*_S_, *I*_D_) parameter space for 
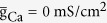
, 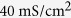 and 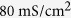. One can find that the threshold value of input *I*_S_ for triggering APs in somatic chamber decreases linearly with dendritic input *I*_D_ (Fig. 5a–c). This is because that introducing positive *I*_D_ results in a depolarizing current transmitted to somatic chamber. Such current increases the level of neuronal excitability and makes it more prone to generate APs to somatic stimulus. Then, the rheobase of *I*_S_ decreases with dendritic input *I*_D_.

Varying Ca^2+^ conductance 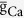 produces no effects on the rheobase of *I*_S_ in the observed range of *I*_D_, which only determines whether there is dendritic Ca^2+^ spike. When blocking Ca^2+^ current (i.e., 
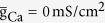
), the dendrite is passive and there is no dendritic Ca^2+^ spike in (*I*_S_, *I*_D_) parameter space. Here the *f_S_*−*I*_S_ curve is always continuous in the range of 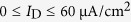 (Fig. 5a), which is generated by a SNIC bifurcation of equilibrium (Fig. 5d). With 
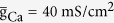
 or 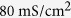, there is dendritic Ca^2+^ spike elicited in (*I*_S_, *I*_D_) parameter space once *I*_D_ reaches a threshold value. Such event causes firing rate *f*_S_ quickly to converge to its plateau level at the onset of *I*_S_ (Fig. 5b and c). Then, the *f*_S_ − *I*_S_ curve becomes discontinuous, which is generated via a SN bifurcation of equilibrium (Fig. 5e). Further, introducing dendritic Ca^2+^ current changes the bifurcation of limit cycle from SNIC to SHO, and makes it occur at another value of *I*_S_ below the bifurcation of equilibrium. Meanwhile, increasing 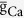 is also able to alter the value of *I*_S_ for causing the SHO bifurcation of limit cycle (Fig. 5e).

**Figure 6:**
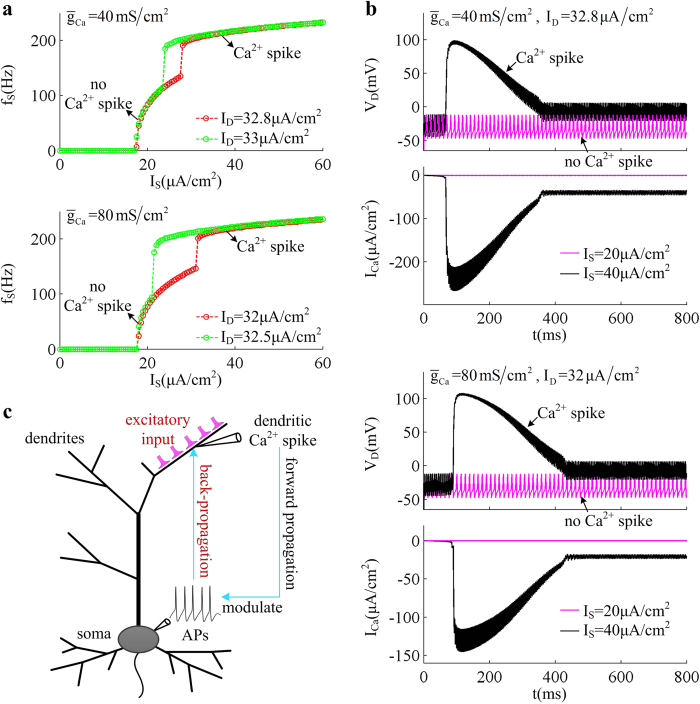
BAC firing evoked by coincident injections to two chambers. **(a**) 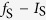 curves of 5D model with 
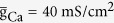
 and 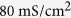. For 
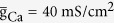
, the value of dendritic input is 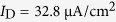 and 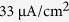. For 
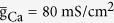
, they are 
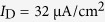
 and 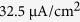. (**b**) Dendritic voltage *V*_D_ and associated Ca^2+^ current *I*_Ca_ for each value of 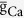. The value of *I*_S_ and *I*_D_ are indicated in each plot. (**c**) Dendritic Ca^2+^ spike is evoked by the interactions between the back-propagating APs and the excitatory inputs received by apical dendrites. The Ca^2+^ spike induced by back-propagating APs is also referred to as BAC firing, which can be propagated to soma to modulate the output APs.

We also find that the threshold values of apical input *I*_D_ for evoking dendritic Ca^2+^ spike in (*I*_S_, *I*_D_) parameter space are significantly lower than those in the absent of *I*_S_. This indicates that the bottom-up input is conducive to the initiation of dendritic Ca^2+^ spike. Particularly, with some moderate values of *I*_D_, the Ca^2+^ current is unable to be activated by *I*_S_ above and close to the bifurcation point of equilibrium (Fig. 6a). Here only somatic input with sufficient intensity can force *V*_D_ to reach the threshold for initiating dendritic Ca^2+^ spike (Fig. 6b). It is known that the firing frequency *f*_S_ is an increasing function of *I*_S_. Thus, the back-propagation of spiking behavior with high frequency contributes to activating Ca^2+^ spike, which is also referred to as BAC firing^17^,^23^,^26^. These results demonstrate that the dendritic Ca^2+^ spike is the outcome of the interaction between back-propagated APs and excitatory synaptic inputs (Fig. 6c). Such prolonged regenerative spikes elicited in apical dendrites can be forward propagated to the AP initiation zone to modulate the final output of the neuron.

## Discussion

Our simulations have shown that injecting current to soma alone makes the 5D model neuron generate continuous input-output relation through a SNIC bifurcation of equilibrium. Here the bottom-up input from soma to dendrite is unable to activate dendritic Ca^2+^ conductance. Thus, varying Ca^2+^ conductance produces no effects on the output APs. Injecting current to apical dendrite alone results in a distinct input-output relation. When blocking Ca^2+^ channel, the input-output relation is still continuous, and the bifurcation structures of equilibrium and limit cycle both remain the same. When there is active Ca^2+^ channel in dendrite, the apical input with sufficient intensity is able to activate Ca^2+^ conductance and trigger a prolonged Ca^2+^ spike. This event boosts depolarized input as it spreads to soma, and facilitates the initiation of somatic APs. Under this condition, the neuron generates a burst of high-frequency APs during the course of dendritic Ca^2+^ spike. Then, the 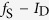 curve becomes discontinuous, and the firing rate quickly reaches a plateau level. These simulations demonstrate that the top-down information received by passive or active dendrite modulates the output APs in a distinct way, which depends critically on the site of synaptic inputs.

The firing rate of the 5D model neuron evoked by conjunct inputs to dendrite and soma is summarized in (*I*_S_, *I*_D_) parameter space. It is shown that simultaneously injecting constant current to two chambers shifts the threshold of 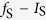 curve to a lower value. When blocking dendritic Ca^2+^ current, the top-down input arriving at apical dendrite only increases the excitability of the neuron and reduces the rheobase of somatic input, which does not alter the shape of input-output relation. For the dendrite with Ca^2+^ channel, the top-down input with sufficient intensity triggers Ca^2+^ spike. This event results in a burst of APs in soma and significantly increases firing frequency, which leads to a discontinuous input-output relation. Here the timing of burst is able to detect whether there are coincident somatic and dendritic inputs. In fact, such burst pattern associated with dendritic Ca^2+^ spike has been observed in previous experimental^26^,^30^,^31^,^32^ and modeling^35^,^37^,^39^,^40^ reports. We have not only reproduced it with a simplified conductance-based model, but also determined the dynamical basis of relevant spike initiation with phase plane analysis. These investigations could contribute to uncovering how the Ca^2+^ activity in apical dendrites participates in neuronal computation.

It is shown that somatic input decreases the threshold value of apical input for triggering dendritic spike. This indicates that the back-propagating APs are conducive to the initiation of dendritic Ca^2+^ spike, i.e., BAC firing occurs. The simulations with our simple 5D model suggest that the generation of BAC firing arises from the interactions between back-propagating spikes and dendritic excitatory input, which is in accordance with previous predictions^17^,^23^,^26^,^27^. During this procedure, the back-propagating APs play a crucial role in connecting two zones of spike initiation, which enables the integration of synaptic inputs to be disturbed in space and time. Meanwhile, BAC firing is a common mechanism for pyramidal neuron to associate conjunct somatic and dendritic inputs^25^. Without BAC firing, the dendritic input has much less effects on the firing behavior than somatic input. Once it occurs, the firing rate and spike timing is dominated by the input received by apical dendrites. Thus, this event completely alters the relative importance of synaptic inputs to the cell. Two-compartment model is the minimum individual unit of the neuron to capture such complex phenomenon. Each chamber has its own mechanism for spike initiation, which enables the neuron to integrate synaptic inputs simultaneously in two separated regions. Thus, it can effectively reproduce the BAC firing. Further, earlier *in vitro* experiment^26^ has predicted that the distal dendritic inputs lead to a higher gain and higher variability of the spike train than somatic input. This phenomenon is missing in our simulations, which is because that the injected currents here are all deterministic and do not include noise. Larkum *et al*.^26^ have shown that the noisy components in current injection are the dominant factor for relevant gain modulation, since they significantly alter the initial slope of input-output relation.

**Figure 7:**
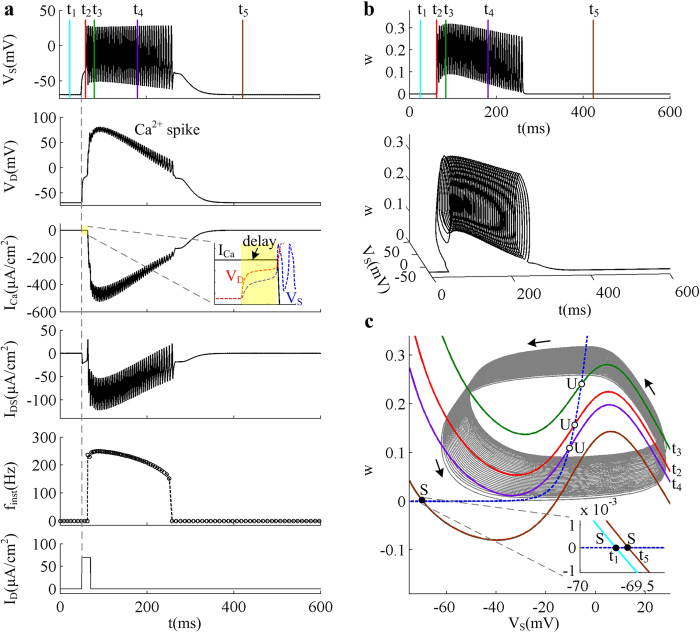
Somatic APs and initiating dynamics evoked by pulse injected at dendritic chamber. (**a**) Sample responses of the 5D model triggered by pulse input *I*_D_ in the cases of 
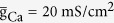
. Somatic voltage *V*_S_, dendritic voltage *V*_D_, dendritic Ca^2+^ current *I*_Ca_, internal current *I*_DS_, and instantaneous firing rate *f*_inst_ are plotted against time. The amplitude of *I*_D_ is 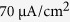 and its duration is 20 ms. **(b)** Plot of K^+^ activation variable *w* against time with 
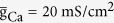
. The spike train recorded in soma is also plotted in 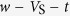 space. Five color lines in panel **(a)** and (**b**) indicate the times at which the nullclines are calculated. (**c**) Two-dimensional phase portraits in 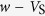 plane. The *V*_S_- and *w*-nullclines are respectively calculated at time point *t*_1_ − *t*_5_ indicated by colored lines in **(a)** and (**b**). Blue dotted line represents *w*-nullcline, and the inverted N-shape lines are the *V*_S_-nullclines at corresponding time point. Gray solid line is the *V*_S_ trajectory, and black arrows indicate its direction. ‘S’ indicates stable fixed point, and ‘U’ indicates unstable fixed point. Somatic injection is 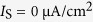.

The dendritic Ca^2+^ spikes in our simulations are triggered by current steps injected to the dendrite. Its roles are discussed in augmenting the influence of dendritic current flowing from the dendrite to the soma over the Na^+^-APs. That is, the influences of relevant Ca^2+^ spike are studied in the presence of background activity. This makes the argument that Ca^2+^ spike augments dendritic current problematic. To determine how dendritic Ca^2+^ spike influences somatic APs by itself, we use apical input to generate a brief pulse to trigger a single dendritic Ca^2+^ spike and repeat above simulations. As expected, the inward current associated with dendritic Ca^2+^ spike provides a strong local depolarization that boosts apical input. The resulting sustained depolarization spreads to the soma and causes a burst of high-frequency APs (Fig. 7). These modulatory effects are similar to that evoked by current steps. It indicates that a Ca^2+^ spike in apical dendrites without additional dendritic input influences the initiation of somatic APs just the same as those with dendritic inputs.

**Figure 8:**
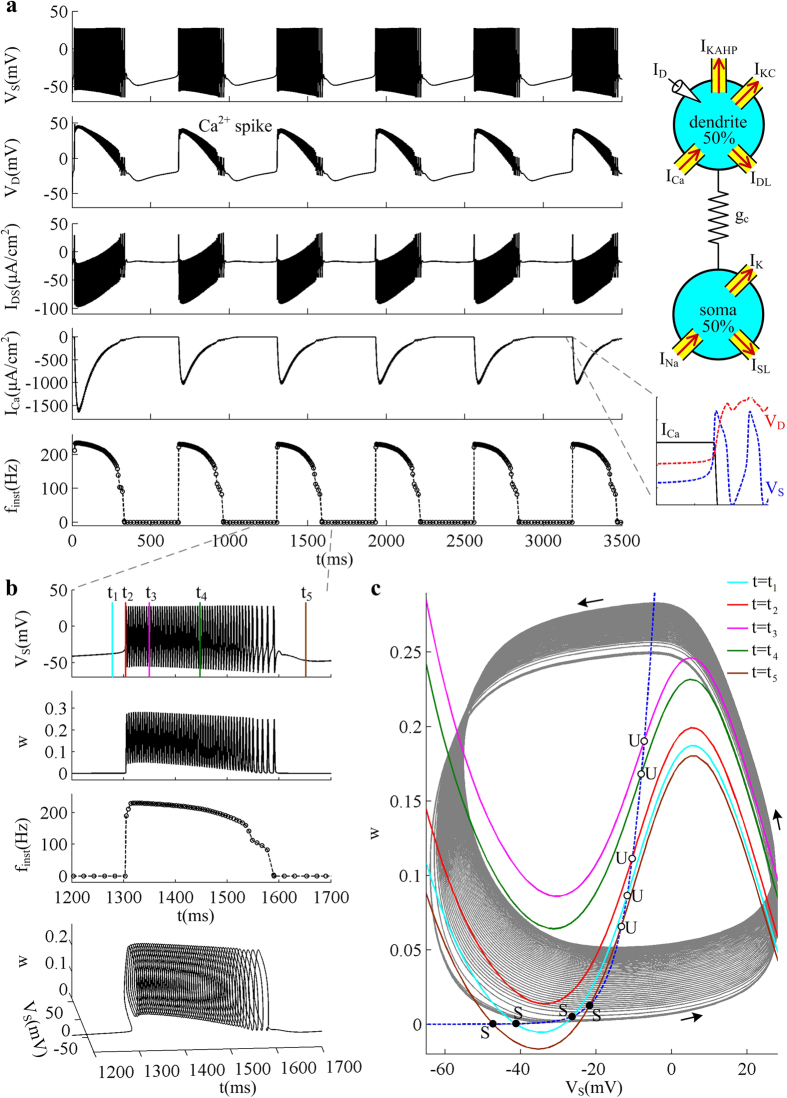
Bursting activities associated with dendritic Ca^2+^ spike in a more biophysically realistic model. (**a**) Schematic of the biophysically realistic model is shown in the top-right panel. The apical input is 
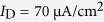
, and somatic input is 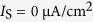. Left panels show the sample responses recorded in its soma. Somatic voltage *V*_S_, dendritic voltage *V*_D_, internal current *I*_DS_, dendritic Ca^2+^ current *I*_Ca_, and instantaneous firing rate *f*_inst_ are plotted against time. The bottom-right close-up shows that the Ca^2+^ conductance is activated when dendritic voltage *V*_D_ exceeds a threshold value. (**b**) Close-up of somatic voltage *V*_S_, K^+^ activation variable *w*, and firing rate *f*_inst_. Bottom panel shows the close-up of *V*_S_ trajectory in 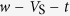 space. Five color lines indicate the times at which the nullclines are calculated. (**c**) Two-dimensional phase portraits in 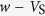 plane. The *V*_S_- and *w*-nullclines are respectively calculated at time point *t*_1_ −*t*_5_ indicated by colored lines in (**b**). Blue dotted line represents *w*-nullcline, and the inverted N-shape lines are the *V*_S_-nullcline at corresponding time point. Gray solid line is the *V*_S_ trajectory, and black arrows indicate its direction. ‘S’ indicates stable fixed point, and ‘U’ indicates unstable fixed point. The maximum conductance of dendritic Ca^2+^ current is 
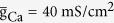
.

Many Ca^2+^-models include the dynamics of intracellular Ca^2+^ concentration 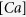
37,38,39,43,49,50,51,52,53, which is tightly related to the Ca^2+^ influx through voltage-gated channels. It has been used in modeling^37^,^39^,^40^ and experimental^15^,^16^ studies to characterize dendritic Ca^2+^ spike. Here we introduce the dynamics of 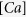 and two types of Ca^2+^-activated K^+^ current to the dendrite of our 5D model (see Methods for model specification). By injecting current step to apical dendrite, we repeat the simulations to test whether dendritic Ca^2+^ spike has effects comparable with those described above. It is shown that the neuron generates periodic bursting behavior to constant apical input after introducing Ca^2+^ concentration and K^+^ currents (Fig. 8a), which makes the difference between burst and tonic spiking more distinguishable. As expected, each dendritic Ca^2+^ spike has similar effects on the initiation of somatic APs in more biophysically realistic model (Fig. 8) and in 5D model (Figs 2 and 7). This suggests that the simplifications inherent in our 5D two-compartment model do not compromise the applicability of our findings to biophysically realistic conditions.

As a common cell type in mammalian brain, pyramidal neurons have been studied with theoretical approaches that incorporate dendritic Ca^2+^ channel in multi-compartmental models^21^,^33^,^34^,^35^,^36^,^37^. These complex models may express more than 10 voltage-gated channels, which are non-homegenously distributed along the somato-dendritic axis. Using biophysically realistic, high-dimensional neuron models is reasonably straightforward. But they may fail to provide greater insights into the mechanism underlying AP initiation than the experiments upon which they are based, since they include so many extraneous details. There are also theoretical studies using simple two-compartment models to describe the Ca^2+^ activity of dendrites for pyramidal cell^38^,^39^,^40^,^41^,^42^,^43^. However, none of them has provided a satisfied interpretation of how dendritic Ca^2+^ spike participates in the initiating dynamics of somatic/axonal APs. Unlike earlier models, our model starts simple and excludes extraneous details, which is made only as complex as required to reproduce the phenomena of interest. It enables one to perform phase plane and bifurcation analysis on how Ca^2+^ spikes initiated in apical dendrites affect the global integration of the neuron. With our simple model, one can visualize and interpret how two-stage integration mode occurs in pyramidal cells. Even so, our predictions and corresponding interpretations require validation with complex models and experiments.

In summary, the current study addresses the importance of Ca^2+^ spike of apical dendrites in affecting the firing behaviors of two-compartment neurons during different sites of current injection. Our simulations provide a deep and interpretable insight into the connection between dendritic Ca^2+^ spike and firing pattern by relating them to somatic AP initiation. Determining how dendritic Ca^2+^ activities and input locations modulate the cellular responses is a pivotal first step toward uncovering how the Ca^2+^ activity of active dendrite participates in neuronal computation. The simplified two-compartment model proposed here is able to capture the complex phenomena of pyramidal neurons in experiments, which could be used to obtain a mechanistic understanding about how relevant circuits participate in cortical computation.

## Methods

Simulations are based on the two-compartmental models of cortical pyramidal neuron, which are the reduced version of PR model. One compartment represents the apical dendritic zone, and the other one represents the basal integration zone around the soma plus axonal initial segment. From apical integration zone, the input signals transmitted to soma can be processed via dendritic Ca^2+^ spike. Two-compartment neuron is the simplest structure to capture such spatial inputs.

Our starting model is derived by introducing a voltage-dependent Ca^2+^ current to the passive dendrite of a simple two-compartment model proposed in our earlier studies^45^,^46^. The right panel of Fig. 1a shows the schematic representation of the two-compartment neuron, which is a 5D model. There are three ionic currents in somatic compartment, which are inward Na^+^ current (*I*_Na_), outward K^+^ current (*I*_k_), and passive leak current (*I*_SL_). Here two active currents, i.e., *I*_Na_ and *I*_k_, are responsible for generating APs. For dendritic chamber, there are two ionic currents, which are inward Ca^2+^ current (*I*_Ca_) and passive leak current (*I*_DL_). The somatic and dendritic chambers are connected by an internal coupling conductance 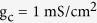. The dynamics of their membrane potential *V*_S_ and *V*_D_ are governed by the following current-balance equations


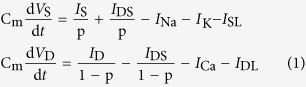


where 
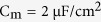
 is the membrane capacitance, 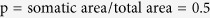 is a morphological parameter. 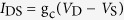 is the internal current that flows through conductance g_c_ and connects two chambers. *I*_S_ and *I*_D_ are two input currents respectively injected at soma and dendrite, which are used to stimulate the neuron.

Three voltage-dependent currents included in somatic chamber are


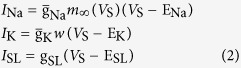


where 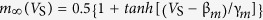 is the steady-state activation function for inward Na^+^ channel. *w* is the activation variable for slow K^+^ current, which is governed by the following differential equation


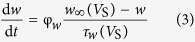


Here 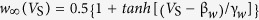 and 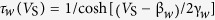 are respectively the steady-state activation function and time constant of this slow current. 
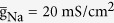
, 
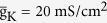
, 
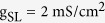
 are the maximum conductances associated with the currents, and 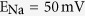, 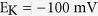, 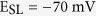 are their relevant reversal potentials. Unless otherwise stated, 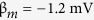, 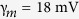, 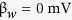, 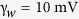, and 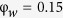.

Two voltage-dependent currents used in dendrite are


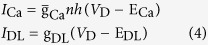


where 
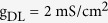
, 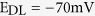, 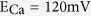, and Ca^2+^ maximum conductance 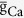 is varied as explained in Results. The dynamics of Ca^2+^ current is governed by an activation variable *n* and an inactivation variable *h*, which are characterized by the following equations


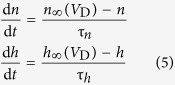



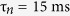
 and 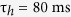 are the time constant of the first-order kinetics for variable *n* and *h*. Their steady-state functions are


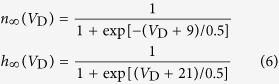


The kinetics of dendritic Ca^2+^ channel here is the same as that described by Larkum *et al*.^26^. Unless otherwise stated, our stimulations are all based on this 5D model.

To make above 5D two-compartment model more biophysically realistic, we introduce the dynamics of intracellular Ca^2+^ concentration 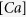 and two types of Ca^2+^-activated K^+^ current to its dendrite (see Fig. 8). The K^+^ channels include short-duration Ca^2+^-dependent K^+^ current (*I*_KC_) and long-duration Ca^2+^-dependent K^+^ current (*I*_KAHP_), which are commonly distributed in the dendrites of pyramidal cells^37^,^39^,^40^. Their activations are both related to the dynamics of 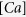. After introducing *I*_KC_ and *I*_KAHP_, the membrane equation for dendritic voltage *V*_D_ becomes


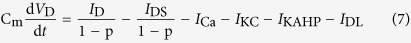


The details of *I*_KC_ and *I*_KAHP_ follow the descriptions by Pinsky and Rinzel^39^, which are 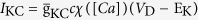, and 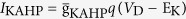. Values of the maximum conductance are 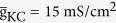 and 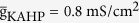. The kinetics of their activation variable *c* and *q* obeys


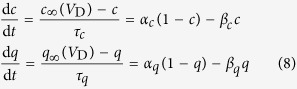


where


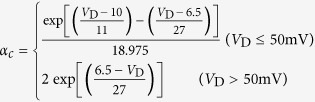



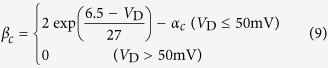



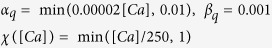


The kinetics for intracellular Ca^2+^ concentration [*Ca*] follows


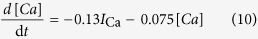


That is, the [*Ca*] is increased proportionally to Ca^2+^ influx *I*_Ca_^37^,^39^. All other parameters in this more biophysically realistic model are the same as in the 5D model.

Note that the dynamics of [*Ca*] is not considered in the simple 5D model. As mentioned in Introduction, dendritic Ca^2+^ spike is the voltage transient caused by the activation of dendritic Ca^2+^ conductance^5^,^6^,^7^,^11^,^23^. It is a local regenerative response involving the positive feedback loop between dendritic voltage and Ca^2+^ influx^6^,^7^. Using a voltage-dependent current *I*_Ca_ is sufficient to reproduce such regenerative dendritic response and its dependence on Ca^2+^ dynamics^26^. By excluding the extraneous details, we propose a biophysical model complex enough to reproduce dendritic Ca^2+^ spike yet simple enough for characterizing its effects on the initiating dynamics of somatic APs. Further, the results in Fig. 8 show that ignoring the dynamics of Ca^2+^ concentration [*Ca*] does not alter our predictions about how dendritic spike affects the initiating dynamics of somatic APs.

Finally, the two-compartment models are integrated in MATLAB using numerical integrator ode23, with a time resolution of 0.01 ms. The phase plane and bifurcation analyses are performed with the publicly available software package XPPAUT^54^.

## Additional Information

**How to cite this article:** Yi, G. *et al*. Action potential initiation in a two-compartment model of pyramidal neuron mediated by dendritic Ca^2+^ spike. *Sci. Rep.* **7**, 45684; doi: 10.1038/srep45684 (2017).

**Publisher's note:** Springer Nature remains neutral with regard to jurisdictional claims in published maps and institutional affiliations.
